# Strategies to Improve the Lipophilicity of Hydrophilic Macromolecular Drugs

**DOI:** 10.1002/adhm.202503721

**Published:** 2025-10-15

**Authors:** Sera Lindner, Stefan Keim, Soheil Haddadzadegan, Odile Fernandez Romero, Katrin Zöller, Gabriel Stern, Ilaria Cesi, Krum Kafedjiiski, Andreas Bernkop‐Schnürch

**Affiliations:** ^1^ Department of Pharmaceutical Technology University of Innsbruck Institute of Pharmacy Center for Chemistry and Biomedicine Innsbruck 6020 Austria; ^2^ Center for Sustainable Materials School of Materials Science and Engineering Nanyang Technological University Singapore 639789 Singapore; ^3^ Thiomatrix Forschungs‐ und Beratungs GmbH Trientlgasse 65 Innsbruck 6020 Austria; ^4^ Department of Pharmaceutical Science Trakia University‐Stara Zagora Stara Zagora 6000 Bulgaria

**Keywords:** conjugation, hydrophobic ion pair, lipidation, lipophilicity, macromolecules, reverse micelles, surfactants

## Abstract

Macromolecular drugs, including peptides, proteins, oligonucleotides, and polysaccharides, have shown remarkable therapeutic potential due to their high specificity, potency, and low toxicity profiles. However, their clinical translation, particularly for oral administration, remains limited by poor bioavailability arising from poor membrane permeability and enzymatic instability. Enhancing the lipophilicity of these molecules is a critical strategy to overcome these challenges, improving their membrane permeability, stability, and pharmacokinetic properties. This review discusses current strategies to improve the lipophilicity of macromolecular drugs, focusing on covalent and non‐covalent lipidation. Covalent lipidation, which involves the conjugation of lipids such as fatty acids or steroids, provides stable chemical modifications that have led to several commercially successful products. However, it also presents regulatory complexities due to the formation of new active pharmaceutical ingredients. In contrast, non‐covalent lipidation methods, such as hydrophobic ion pairing and reverse micelle formation, offer reversible alternatives that preserve the native structure of the drug, simplify regulatory procedures, and allow flexible tuning of delivery properties. Notably, reverse micelle systems demonstrate superior performance compared to hydrophobic ion pairs, particularly in enhancing the lipophilicity of larger, more complex macromolecules. While lipidation strategies have significantly advanced the field, substantial challenges remain, especially in achieving consistent bioavailability and translating preclinical success into clinical efficacy. Future progress will require innovative ideas and the integration of emerging technologies to fully unlock the potential of lipidated macromolecular therapeutics.

## Graphical abstract

Hydrophilic macromolecular drugs can be successfully lipidized by covalent attachment of lipids, by hydrophobic ion pairing with negatively or positively charged surfactants, and by dry or wet reverse micelle formation. Lipophilicity enhancement of hydrophilic macromolecules has several benefits including stability and bioavailability improvement.

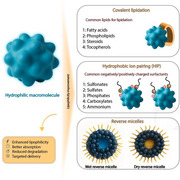

## 1 Introduction

Macromolecular drugs, including peptides, proteins, oligonucleotides, and polysaccharides, have demonstrated great promise as novel therapeutics in the treatment of a wide range of diseases. Compared to small molecule drugs, these large molecules offer several advantages, including enhanced potency, target specificity, reduced toxicity, and lower incidence of drug–drug interactions.^[^ ^1^ ^]^ Notably, the market for peptide‐based therapeutics has been constantly growing since the late 1990s, with an estimated 140 peptide drugs currently undergoing clinical trials and 500 therapeutic peptides in pre‐clinical development.^[^ ^2^, ^3^ ^]^ Structural and functional modifications using chemical techniques have been used to generate compounds with higher affinity, improved enzymatic stability, and efficacy compared to the parent peptide.^[^ ^1^, ^4^ ^]^ Gene therapy has also emerged as a powerful platform, enabling targeted interventions against disease‐causing genes and paving the way for precise, personalized treatments. Therapeutics based on small interfering RNA (siRNA) and antisense oligonucleotides (ASOs) have been developed to downregulate gene expression, driving rapid growth in this field and leading to several approved products.^[^ ^5^ ^]^ Chemical modifications of therapeutic oligonucleotides further enhance their stability and activity.^[^ ^5^, ^6^ ^]^ Among therapeutic polysaccharides, heparin and its low molecular weight derivatives are the most prominent. Initially developed as anticoagulants, these molecules have since demonstrated potential in antitumor, anti‐inflammatory, and antiviral applications, necessitating ongoing optimization for broader clinical use.^[^ ^7^ ^]^

Despite these advances, macromolecular drugs, particularly when administered orally, face significant challenges in bioavailability. Their large molecular size and hydrophilic nature impede effective absorption.^[^ ^8^ ^]^ The lipophilicity of a drug or drug candidate is a critical factor in developing drug delivery systems, as it influences absorption, distribution, metabolism, excretion, and toxicity by affecting its pharmacokinetic, pharmacodynamic, and toxicological profiles.^[^ ^9^ ^]^ Research indicates that adequate lipophilicity is essential for developing drug candidates that achieve successful commercialization. Most importantly, macromolecular drugs must penetrate the lipid bilayer of cellular membranes, including enterocytes, to be absorbed effectively and delivered to their target sites for therapeutic action. Consequently, drug molecules should possess lipophilic properties to ensure sufficient permeability through biological membranes, optimal distribution, and plasma protein binding for enhanced stability.^[^ ^10^ ^]^ However, most therapeutic macromolecules are highly hydrophilic, creating significant challenges for oral absorption and bioavailability. Additional limitations include short half‐lives, enzymatic instability, limited tissue distribution, and rapid systemic clearance.^[^ ^11^ ^]^ To address these issues and enhance therapeutic efficacy, strategies that improve the lipophilicity of macromolecular drugs are critical. These strategies are broadly classified into covalent and non‐covalent lipidation approaches, each with distinct advantages and limitations.

Covalent lipidation involves the chemical attachment of lipid moieties to the macromolecular drug and has been particularly successful for peptide drugs, as evidenced by marketed products such as liraglutide (Victoza)^[^ ^12^ ^]^ and insulin detemir (Levemir),^[^ ^13^ ^]^ where pharmacokinetic and pharmacodynamic properties have been favorably altered. Alternatively, non‐covalent lipidation strategies exploit electrostatic and hydrophobic interactions between hydrophobic moieties and functional groups of macromolecules. Emerging methods include hydrophobic ion pairing and the use of reverse micelle formulations. While hydrophobic ion pairing significantly improves membrane permeability and oral bioavailability, reverse micelle formation is less dependent on the charge of the macromolecule, broadening its applicability.

In this review, we summarize current strategies aimed at enhancing the lipophilicity of macromolecular drugs to overcome limitations related to stability, activity, and delivery. We also critically discuss covalent and non‐covalent lipidation approaches, highlighting their respective benefits and challenges.

## 2 Lipid Conjugation

One widely used approach to increase the lipophilicity of hydrophilic macromolecular drugs is the covalent conjugation of hydrophobic compounds, such as lipids. This lipidation process is not only commonly used in pharmaceutical development to optimize drug properties but is also a naturally occurring modification in the body.^[^ ^2^, ^14^ ^]^ Various types of lipids – such as fatty acids, steroids, alkanes, terpenes, tocopherol, and phospholipids – can be used in macromolecule lipidation.^[^ ^14^ ^]^

Generally, lipid conjugation enhances the delivery of macromolecules by increasing hydrophobicity and membrane affinity, facilitating cellular uptake and endothelial permeability. Two principal mechanisms affect lipidation‐mediated uptake: endocytosis and direct translocation.^[^ ^15^ ^]^ These processes often occur in parallel, with the dominant pathway determined by lipid chain length, degree of modification, aggregation state, and the cellular context. Increased membrane association through insertion into the lipid bilayer, driven by the lipid moiety, prolongs residence time on the cell surface, enhancing capture by the endocytic machinery. Lipidated molecules preferentially partition into cholesterol‐ and sphingolipid‐rich membrane microdomains (lipid rafts), promoting caveolae‐ and raft‐mediated endocytosis through clustering and recruitment of adaptor proteins. Additionally, lipid anchors can locally perturb membrane packing and induce curvature, lowering the energetic barrier for vesicle formation and favoring macropinocytosis. Self‐assembly into micelles or nanoparticles further facilitates uptake, with size‐dependent endocytic pathways: smaller particles (<150 nm) typically utilize clathrin‐ or caveolae‐mediated endocytosis, whereas larger assemblies engage macropinocytosis or phagocytosis. Direct translocation also contributes to cellular uptake, particularly for smaller, cationic, or single‐lipid molecules. In this process, the lipid tail inserts into the bilayer, transiently disrupting membrane integrity and enabling cytosolic delivery without vesicular trafficking. While less efficient for larger complexes, this pathway is important when rapid cytosolic access is needed.

To illustrate the translational impact of lipid conjugation, **Table** **1** summarizes macromolecular drugs that have successfully reached the market. These examples demonstrate the clinical feasibility and therapeutic benefits of such modifications in enhancing drug performance. Complementing this, **Table** **2** lists drugs currently in clinical development, highlighting ongoing efforts to expand the pipeline of next‐generation macromolecular therapeutics.

**Table 1:** Overview of marketed lipidized macromolecular drugs and their key characteristics.

Name	Trade name (Company)	Molecule type	Lipidation	Mode of action	First market launch
Insulin detemir	Levemir (Novo Nordisk)	Peptide	Myristic acid (C14)	Long‐acting insulin derivative	2004 (EU)
Insulin degludec	Tresiba (Novo Nordisk)	Peptide	Palmitic acid (C16)	Long‐acting insulin derivative	2013 (UK/DK)
Insulin icodec	Awiqli (Novo Nordisk)	Peptide	1,20‐icosanedioic acid (C20)	Long‐acting insulin derivative	2025 (JP)
Tirzepatide	Mounjaro (Eli Lilly)	Peptide	1,20‐icosanedioic acid (C20)	GIP/GLP‐1 agonist	2022 (US/EU)
Semaglutide	Ozempic (Novo Nordisk)	Peptide	Stearic acid (C18)	GLP‐1 agonist	2017 (US)
Liraglutide	Victoza (Novo Nordisk)	Peptide	Palmitic acid (C16)	GLP‐1 agonist	2009 (EU)
Somapacitan	Sogroya (Novo Nordisk)	Protein	Palmitic acid (C16) tetrazole derivative	Growth hormone analogue	2020 (US)
Imetelstat	Rytelo (Geron)	Oligonucleotide	Palmitic acid (C16)	Telomerase inhibitor	2024 (US)
Tesamorelin	Egrifta SV (Theratechnologies)	Peptide	Hexanoic acid (C6)	GNRH analogue	2010 (US)
Anidulafungin	Eraxis (Pfizer)	Peptide	C5‐hydroxyhexyl‐linked terphenyl	Antimycotic	2006 (US)
Daptomycin	Cubicin (MSD)	Peptide	Decanoic acid (C10)	Antibiotic	2003 (EU)
Dalbavancin	Xydalba (Allergan)	Glycopeptide	Different alkyl sidechains	Antibiotic	2015 (EU)
Telavancin	Vibativ (Cumberland Pharmaceuticals)	Glycopeptide	Decylaminoethyl side chain	Antibiotic	2009 (US)
Teicoplanin	Targocid (Sanofi)	Glycopeptide	Different fatty acids	Antibiotic	1988 (EU)

**Table 2:** Overview of lipidized macromolecular drugs currently in clinical development. When multiple clinical trials exist, only the number and the start date of the most recent trial in the highest phase (Phase 1–3) are reported.

Drug	Molecule type	Phase(s)	Status	Clinical trial	Sponsor	Mode of action	Conditions	Lipidation	Reference
Pemvidutide	Peptide	2	Ongoing	NCT07009860	Altimmune	GLP‐1/ Glucagon receptor agonist	Alcohol Liver Disease	Stearic acid (C18)	[16, 17]
Cagrilintid	Peptide	1–3	Ongoing	NCT07011667	Novo Nordisk	Amylin‐Analogue	Multiple metabolic conditions	1,20‐icosanedioic acid (C20)	[18]
Survodutide	Peptide	1–3	Ongoing	NCT06632444	Boehringer Ingelheim	GLP‐1/ Glucagon receptor agonist	Multiple metabolic conditions	1,20‐octadecanedioic acid (C18)	[19]
Mazdutide	Peptide	1–4	Ongoing, already marketed in China	NCT06931028	Innovent Biologics and Eli Lilly	GLP‐1/ Glucagon receptor agonist	Multiple metabolic conditions	1,20‐icosanedioic acid (C20)	[20, 21]
Retatrutide	Peptide	1–3	Ongoing	NCT07035093	Eli Lilly	GLP‐1/ GIP/Glucagon receptor agonist	Multiple metabolic conditions	1,20‐icosanedioic acid (C20)	[22, 23]
AZD6234	Peptide	1–2	Ongoing	NCT07017179	AstraZeneca	Amylin analogue	Multiple metabolic conditions	Fatty acid derivative	[24, 25]
Petrelintide	Peptide	1–2	Ongoing	NCT06926842	Zealand Pharma	Amylin analogue	Multiple metabolic conditions	1,20‐icosanedioic acid (C20)	[26]
Dapiglutide	Peptide	1–2	Ongoing	NCT05788601	University Hospital, Gentofte, Copenhagen	GLP‐1/GLP‐2 receptor agonist	Obesity	1,20‐octadecanedioic acid (C18)	[27]
PYY1875	Peptide	2	Completed	NCT04969939	Novo Nordisk	Peptide YY analogue	Obesity	Fatty diacid derivative	[28, 29]
LIPO‐5	Peptide	1	Completed	NCT02038842	ANRS	Lipopeptide vaccine	HIV	Palmitic acid (C16)	[30, 31]
Ecnoglutide	Peptide	1–3	Ongoing	NCT05680129	Hangzhou Sciwind Biosciences	GLP‐1 agonist	Multiple metabolic conditions	1,20‐octadecanedioic acid (C18	[32, 33]
PZ‐128	Peptide	2	Completed	NCT02561000	Tufts Medical Center	PAR1 antagonist	Vascular diseases	Palmitic acid (C16)	[34, 35]
ELI‐002 7P	Peptide	1–2	Ongoing	NCT05726864	Elicio Therapeutics	mKRAS vaccine	mKRAS cancer types	Stearic acid (C18) diester derivative	[36, 37]

### 2.1 Conjugation of Fatty Acids

**Figure 1:**
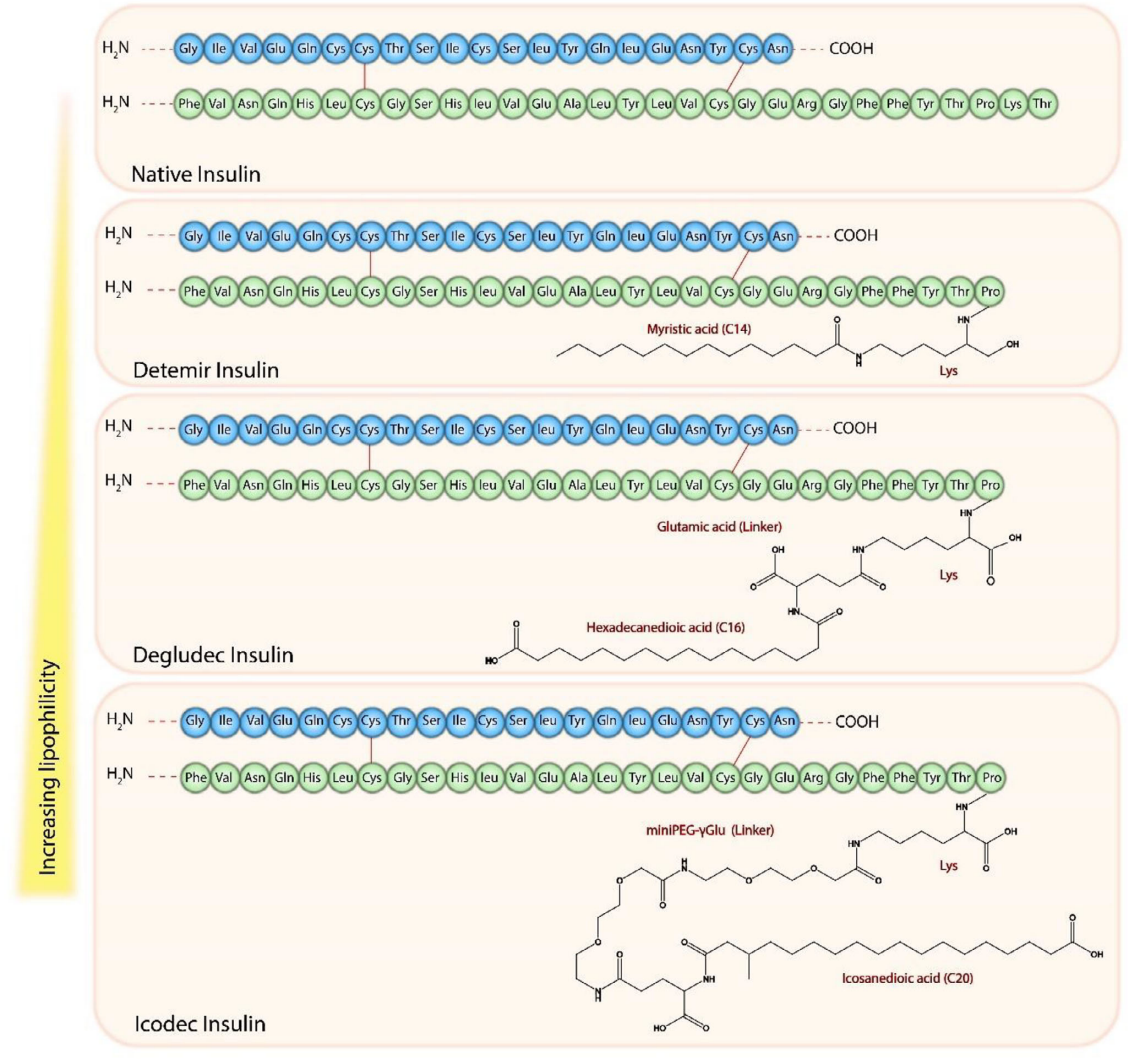
Structure of native human insulin, insulin detemir, insulin degludec, and insulin icodec.

Well‐known examples of fatty acid modified peptides are long‐acting (basal) insulins (**Figure** **1**) which are administered once‐daily or even once‐weekly for the treatment of diabetes mellitus.^[^ ^13^ ^]^ This modification enhances their lipophilicity and increases their reversible binding to circulating serum albumin, resulting in prolonged activity compared to native and rapid‐acting (bolus) insulins, which have short half‐lives of 4–6 min.^[^ ^13^ ^]^ Additionally, recent phase 3 clinical trials (ONWARDS) demonstrated insulin icodec´s superior efficacy compared to once‐daily insulins like insulin glargine and insulin degludec.^[^ ^38^, ^39^ ^]^

**Figure 2:**
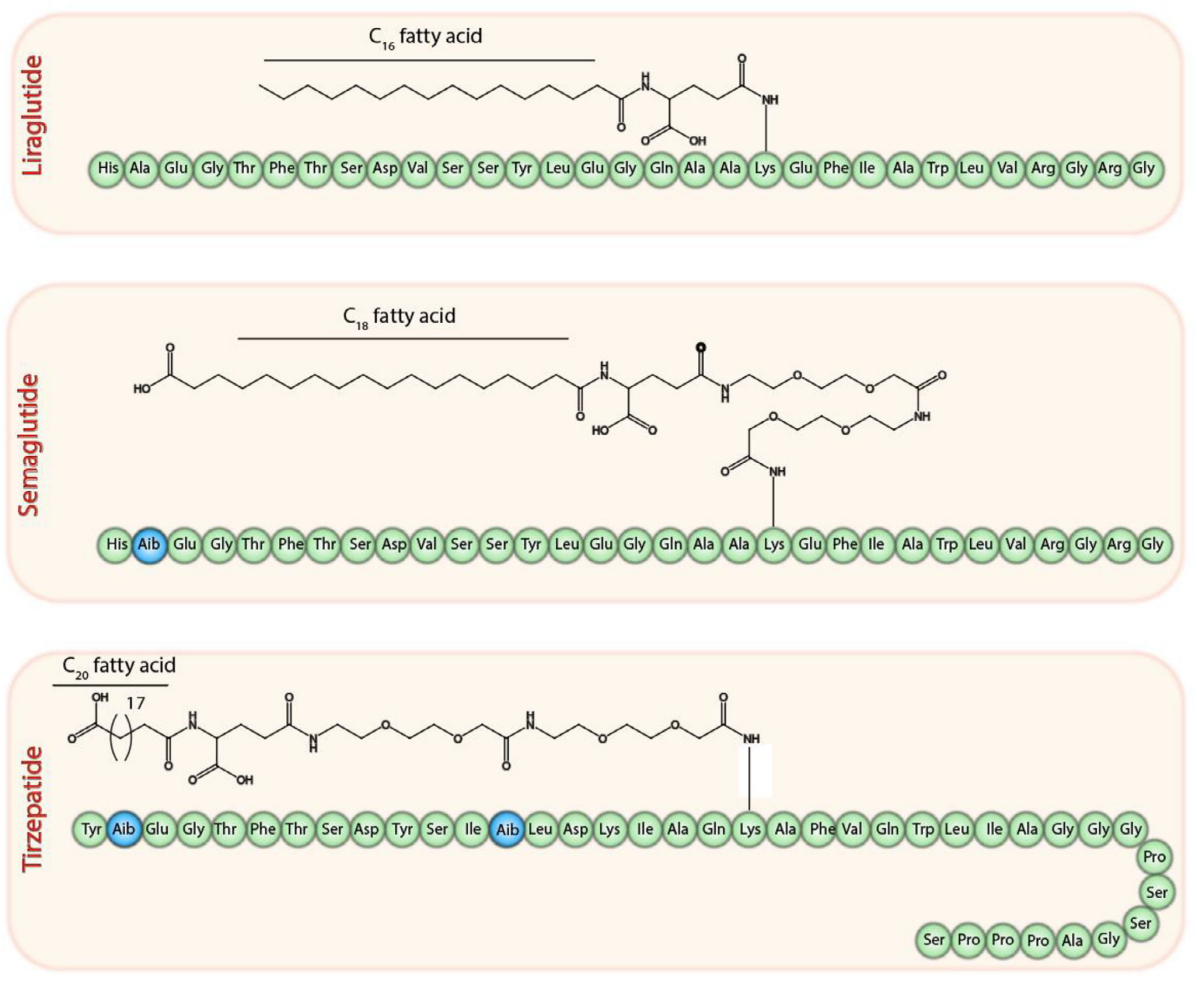
Structure of liraglutide, semaglutide, and tirzepatide.

Similarly, fatty acid conjugation has been used to create effective glucagon‐like peptide 1 (GLP‐1) agonists like liraglutide, semaglutide, and tirzepatide (**Figure** **2**, **Table** **3**). These modifications extend their half‐lives by binding to albumin.^[^ ^12^ ^]^ While native GLP‐1 has a short half‐life of 1.5 min, liraglutide extends it to 13 h, and semaglutide and tirzepatide allow for weekly dosing with roughly 7‐day half‐lives.^[^ ^40^, ^41^ ^]^ Ongoing research aims to improve albumin binding affinity, half‐life, and stability using new or modified fatty acids.^[^ ^42^, ^43^, ^44^, ^45^, ^46^ ^]^ Dual fatty acid conjugation, especially with dodecanoic acid, shows promise in surpassing current GLP‐1 agonist performance.^[^ ^43^ ^]^ Due to fatty acid conjugation, however, GLP‐1 agonists tend to oligomerize and to form fibrils. Liraglutide exhibits, for example, pH and concentration‐dependent aggregation forming 7 and 13‐mers and even fibrils.^[^ ^47^ ^]^

**Table 3:** Comparison of structural characteristics, pharmacological properties, advantages, and disadvantages of liraglutide, semaglutide, and tirzepatide.

Feature	Liraglutide	Semaglutide	Tirzepatide
Fatty Acid Chain	C16 (palmitic acid)	C18 diacid (stearic acid derivative)	C20 diacid (eicosanedioic acid)
Conjugation Site	Lys26	Lys26	Lys20
Linker Type	γ‐glutamic acid spacer	γ‐glutamic acid linker with two polyethylene glycol (PEG) spacers	Glutamic acid linker with two (2‐(2‐aminoethoxy)ethoxy)acetic acid (AEEA) spacers
Modified Amino Acids	None	Substitution of alanine with α‐aminoisobutyric acid (Aib) at position 8 to prevent DPP‐4 degradation	Multiple substitutions to enhance stability and receptor activity; designed as a dual agonist for GIP and GLP‐1 receptors
Half‐Life	≈13 h	≈165–184 h (≈7 days)	≈5 days
Receptor Activity	GLP‐1 receptor agonist	GLP‐1 receptor agonist	Dual agonist for GIP and GLP‐1 receptors
Advantages	‐ Established safety profile ‐ Effective glycemic control	‐ Extended half‐life allows for once‐weekly dosing ‐ Superior weight loss efficacy compared to liraglutide	‐ Superior glycemic control and weight loss compared to semaglutide ‐ Potential benefits in insulin sensitivity and lipid metabolism
Disadvantages	‐ Requires daily injections ‐ Shorter half‐life compared to newer agents	‐ Potential for gastrointestinal side effects ‐ Higher cost	‐ Long‐term safety data still emerging ‐ Complex structure may pose manufacturing challenges

Fatty acylation is also used in oligonucleotide therapeutics to enhance stability and tissue accumulation. Unconjugated small interfering ribonucleic acid (siRNA) and antisense oligonucleotides (ASO) usually accumulate in the liver, kidneys and spleen, leading to rapid elimination (half‐life ≈5 min). This limits their effective distribution to extrahepatic target tissues.^[^ ^48^, ^49^ ^]^ Conjugating these RNA therapeutics with fatty acids improves their association with serum albumin and lipoproteins, enhancing retention and tissue distribution.

For an overview of recently published studies, we refer to different reviews.^[^ ^48^, ^50^, ^51^, ^52^ ^]^ So far, various fatty acids (C12‐C22) have been tested, showing promising efficacy and toxicity profiles.^[^ ^48^, ^50^, ^51^, ^53^, ^54^, ^55^ ^]^ Furthermore, siRNA and ASO could successfully be delivered to extrahepatic targets, including the brain,^[^ ^56^, ^57^, ^58^, ^59^ ^]^ skeletal and cardiac muscles^[^ ^60^, ^61^, ^62^ ^]^ as well as placenta,^[^ ^63^ ^]^ opening new therapeutic opportunities. However, higher lipophilicity might not always be beneficial regarding toxicity. For example, less hydrophobic conjugates improved silencing in glioblastoma cells while maintaining a better toxicity profile, whereas more hydrophobic conjugates exhibited neurotoxicity.^[^ ^56^ ^]^

On the other hand, fatty acylation aims to increase the overall lipophilicity of therapeutic macromolecules, leveraging lipophilicity for therapeutic effects, unlike prior examples that bind with serum compounds to enhance performance. Increasing lipophilicity benefits antimicrobial peptides (AMP) by improving their interaction with bacterial membranes, thus boosting their antimicrobial effect.^[^ ^64^ ^]^ Various natural and synthetic AMP have been conjugated with fatty acids to improve their activity. These peptides are otherwise susceptible to degradation by proteolytic enzymes, physiological salts, and pH conditions.^[^ ^65^, ^66^ ^]^ The effectiveness of lipidized AMP depends on the fatty acid chain length. Studies indicated that medium‐chain fatty acids generally provided optimal antimicrobial activity.^[^ ^66^, ^67^, ^68^, ^69^, ^70^ ^]^ However, longer‐chain fatty acids like palmitic (C16), stearic (C18), and arachidic acid (C20) reduced antimicrobial potency due to self‐assembly into micelles or aggregates.^[^ ^68^, ^71^ ^]^ These modifications, while enhancing stability against proteolysis and serum degradation, also increased cytotoxicity by interacting non‐selectively with both bacterial and eukaryotic membranes.^[^ ^66^, ^69^, ^71^, ^72^ ^]^ Therefore, toxicity profiles must be carefully evaluated. Lipidized AMP also has potential as anticancer agents due to their ability to disrupt lipid bilayers, which is useful for targeting cancer cells.^[^ ^65^ ^]^

Furthermore, fatty acid conjugation aims to enhance the lipophilicity of polysaccharides like heparin, aiding their incorporation into lipophilic carriers for oral administration. Additionally, lipidized heparin derivatives not only improve oral bioavailability but may also enhance binding to hydrophobic surfaces on extracorporeal devices that are heparin‐coated to maintain hemocompatibility.^[^ ^73^ ^]^ Paliwal et al. found that longer fatty acid chains (C18 > C16 > C14) correlated with higher bioavailability and greater bioactivity of low molecular weight heparin (LMWH).^[^ ^74^ ^]^ **Table** **4** provides a summary of representative studies on fatty acid‐conjugated macromolecular drugs, detailing their respective fatty acid conjugates and the beneficial effects of increased lipophilicity.

**Table 4:** Exemplary studies improving the lipophilicity of macromolecular drugs via fatty acid conjugation.

Macromolecule type	Drug	Fatty acid	Effect of increased lipophilicity	Reference
Peptide	Insulin detemir	Myristoyl acid (C14)	Reversible binding to serum albumin, prolonged activity enabling once‐daily treatments	[13]
Peptide	Insulin degludec	Palmitic acid (C16)		[13]
Peptide	Insulin icodec	1,20‐icosanedioic acid (C20)	Reversible binding to serum albumin, significantly increased half‐life of 196 h, allowing for once‐weekly administration	[75]
Peptide	Liraglutide	Palmitic acid (C16)	Reversible binding to serum albumin, increased half‐life of 13 h	[12, 40, 41]
Peptide	Semaglutide	Stearic acid (C18)	Reversible binding to serum albumin, significantly increased half‐life of 7 days for weekly administration	[12, 40, 41]
Peptide	Tirzepatide	Eicosanedioic acid (C20)		[12, 40, 41]
Peptide	Leuprolide	Saturated C18‐fatty acids	Prolonged half‐life, improvement in permeability, and biological activity	[76, 77, 78]
Peptide	Human interleukin 2	Octadecanoic diacid		[79]
Peptide	Pep19‐4LF	Undecanoic acid (C11)	Enhanced antimicrobial activity	[67]
Peptide	AKK	Lauric acid (C12), myristic acid (C14)		[80]
Peptide	Anoplin	Lauric acid (C12)		[66, 69]
Peptide	Melittin	Caprylic acid (C8)		[70]
Peptide	CGA‐N9	Caprylic acid (C8)	Improved antifungal effects against *Candida albicans*, biofilm inhibition, and eradication of pre‐formed biofilm	[81]
Peptide	Figainin 1	C10	Enhanced stability, proteolytic resistance, and antiproliferative activity due to effective interaction with cancer cell membranes	[82]
Peptide	CAMEL	C12		[71]
RNA	siRNA	Palmitic acid (C16)	Enhancement in silencing efficacy	[55, 60, 62, 83]
RNA	siRNA	Docosanoic acid (C22)		[61, 63]
RNA	siRNA	Eicosapentaenoic acid (C20:5)		[56]
RNA	siRNA	Docosahexanoic acid (C22:6)		[59]
Polysaccharide	Heparin	Acyl hydrazides of C8, C10, C12 and C18 or a mixture of C12 and C18	Enhanced hydrophobic interaction with phenyl‐Sepharose columns for longer stearyl hydrazide or combined lauryl and stearyl hydrazides	[73]
Polysaccharide	LMWH	Myristic (C14), palmitic (C16) and stearic (C18) acid	3.6‐fold higher oral bioavailability compared to unconjugated LMWH	[74]

### 2.2 Conjugation of Steroids

Conjugation of steroids presents another promising strategy to improve the lipophilicity and activity of hydrophilic macromolecules, effectively addressing common issues like low stability and inefficient delivery. For siRNA, conjugation of cholesterol is an effective solution, similar to the conjugation of fatty acids discussed previously. This modification increases siRNA lipophilicity, enabling binding to natural carrier proteins, which leads to effective delivery to both liver and extrahepatic tissues with longer half‐lives.^[^ ^84^ ^]^ Additionally, lipidation with cholesterol permits siRNA self‐delivery without needing additional formulations or mechanical delivery systems, enhancing cellular uptake and sustained gene silencing.^[^ ^85^, ^86^ ^]^ This process results in siRNA rapidly associating with plasma membranes through lipophilic interaction and cholesterol intercalation into the membranes.^[^ ^87^ ^]^ For example, Woller et al. investigated self‐delivering siRNA‐cholesterol conjugates promoting axon regeneration in rats after optic nerve injuries.^[^ ^86^ ^]^ Phio Pharmaceuticals developed INTASYL technology using cholesterol‐conjugated, chemically modified siRNA for treating cancer, dermatological, and ophthalmological diseases,^[^ ^85^, ^88^ ^]^ with PH‐762 being currently tested in phase 1 trials (NCT 0 601 4086).^[^ ^89^ ^]^ Cholesterol‐conjugated siRNA therapeutics have potential for treating various conditions. They target inflammatory cytokines in liver macrophages for nonalcoholic hepatic diseases^[^ ^90^ ^]^ or the primary lactate transporter gene MCT1,^[^ ^91^ ^]^ silence myostatin for muscular disorders^[^ ^92^ ^]^ and reduce placental tyrosine kinase sFLT1 for preeclampsia.^[^ ^63^, ^93^ ^]^ For comprehensive studies, we refer to other reviews.^[^ ^48^, ^50^, ^84^, ^88^ ^]^ While cholesterol conjugation offers significant benefits, it also has drawbacks compared to other lipophilic moieties like fatty acids or α‐tocopherol. Cholesterol conjugates can cause increased immune stimulation and dose‐limiting cytotoxicity.^[^ ^62^, ^63^ ^]^

Cholesterol‐modified antiviral fusion inhibition peptides, including those against human immunodeficiency virus (HIV),^[^ ^94^ ^]^ influenza virus,^[^ ^95^, ^96^ ^]^ Ebola virus,^[^ ^97^, ^98^ ^]^ and other viruses,^[^ ^99^ ^]^ benefit from cholesterol´s ability to integrate into cell membranes via lipid raft interactions. This elevated peptide concentration at the membrane and promoted effective fusion on both the cell surface and intracellularly, overcoming potency limitations observed with unmodified peptides.^[^ ^94^, ^95^, ^100^ ^]^ The recent COVID‐19 pandemic has highlighted the need for effective antiviral therapeutics against SARS‐CoV‐2. Cholesterol‐modified lipopeptides have shown significant activity enhancement,^[^ ^101^, ^102^, ^103^ ^]^ potentially expanding to other viruses, offering a promising approach for combating future pandemics.^[^ ^100^ ^]^

Beyond cholesterol, bile acids have also been explored as effective conjugation partners. For example, conjugating GLP‐1 agonists with mono‐, bis‐ and tetra‐deoxycholic acid (DOCA) has resulted in a notable increase in cellular permeability and oral bioavailability.^[^ ^104^ ^]^ This improvement is largely due to enhanced intestinal uptake via the apical sodium‐dependent bile acid transporters (ASBT), which bind conjugated DOCA and facilitate transporter‐mediated endocytosis, thus avoiding lysosomal degradation. Permeation studies demonstrated that DOCA conjugates achieved a 90‐fold higher permeability compared to exenatide, reflected by a significantly improved oral bioavailability (up to 8.6%) in vivo. Furthermore, bile acids have also been utilized to modify heparin and LMWH, as already reviewed by others.^[^ ^105^, ^106^, ^107^ ^]^ Similarly, conjugating heparin with DOCA tremendously improved its intestinal absorption and oral bioavailability, achieving levels up to 33.5% in rats, likely due to ASBT‐mediated cellular uptake.^[^ ^108^ ^]^ Interestingly, conjugation with a combination of taurocholic acid and tetrameric DOCA resulted in the enhancement of antiproliferative effects of heparins, inhibiting tumor growth and metastasis by inhibiting tumor growth factors, opening new possibilities in effective cancer therapy.^[^ ^109^, ^110^ ^]^

### 2.3 Conjugation of Other Lipids

In the field of siRNA research, various lipophilic moieties have been explored as conjugation partners, including phospholipids,^[^ ^111^ ^]^ sphingolipids,^[^ ^112^ ^]^ lipidic polymers, large hydrophobic adamantane‐based groups and twisted intercalating nucleic acids.^[^ ^113^ ^]^ Although less prevalent compared to fatty acid or cholesterol conjugation and necessitating further studies, the most extensively investigated conjugates are alkyl‐, terpene‐, and vitamin‐conjugates.

For alkyl chain‐modified siRNA, attachment of a 2´‐O‐hexadecyl moiety enabled efficient silencing in various extrahepatic organs, including the central nervous system, eyes, and lung for at least 3 months, showing promise for new therapeutic areas.^[^ ^114^ ^]^ Alnylam Pharmaceuticals completed phase 1 trials for their 2´‐O‐hexadecyl‐conjugated siRNA therapeutic, ALN‐APP, which targets Alzheimer's disease and cerebral amyloid angiopathy. Phase 2 trials are underway and expected to conclude in late 2029 (NCT06393712).^[^ ^115^, ^116^ ^]^

In another approach, the triterpene squalene was conjugated to siRNA designed to target the RET/PTC1 fusion oncogene, implicated in papillary thyroid carcinoma.^[^ ^117^ ^]^ In vivo studies demonstrated a 70% reduction in tumor growth and 80% gene silencing, accompanied by decreased protein concentration. This conjugation significantly increased the lipophilicity of siRNA, enabling spontaneous self‐assembly into nanoparticles. Depending on the molecular design, these amphiphiles may organize into diverse nanostructures such as micelles, vesicles, nanofibers, or other supramolecular aggregates. The formation of such assemblies can alter the pharmacological behavior of the parent drug. For instance, self‐assembled micelles can enhance apparent solubility and provide protection against enzymatic degradation, while vesicular or fibrillar assemblies may function as depots enabling sustained release. Their nanoscale properties also modulate biodistribution, circulation half‐life, and tissue penetration. In addition to these protective effects, self‐assembled structures may directly enhance biological activity, for example, by facilitating receptor interactions or improving uptake through endocytic pathways. At the same time, the propensity for aggregation introduces challenges: uncontrolled self‐assembly can reduce reproducibility, complicate pharmacokinetics, or increase immunogenicity.^[^ ^118^, ^119^ ^]^

Additionally, conjugation was expanded to an even more hydrophobic polyisoprenoid, solanesol, via copper‐free click chemistry to enhance siRNA stability. This approach targeted the TMPRSS2‐ERG fusion oncogene associated with prostate cancer and demonstrated in vivo efficacy, though it did not surpass the performance of squalene‐conjugation.^[^ ^120^ ^]^

Several studies have confirmed the efficacy of tocopherol conjugation in siRNA and ASO, demonstrating enhanced hepatic delivery and superior apolipoprotein B gene silencing compared to cholesterol‐conjugates.^[^ ^121^, ^122^ ^]^ Moreover, α‐tocopherol conjugation facilitated siRNA delivery to the brain, targeting β‐amyloid precursor protein cleaving enzyme 1 (BACE1), a key factor in Alzheimer's disease. Additional conjugation with lipoproteins, leveraging lipophilic interactions with tocopherol, further improved delivery to neurons and glial cells via lipoprotein receptor‐mediated endocytosis. This strategy resulted in a substantial 60–70% reduction in BACE1 gene expression, demonstrating the potential of lipid‐based modifications for enhancing RNA‐based therapeutics in neurodegenerative diseases.^[^ ^123^ ^]^

Collectively, these lipid conjugation strategies significantly enhance the lipophilicity, stability, and therapeutic efficacy of macromolecular drugs, paving the way for more effective treatments across various medical fields.

## 3 Interaction with Surfactants

### 3.1 Hydrophobic Ion Pairing

Hydrophobic ion pairing describes a technique by which the solubility profile of ionizable drugs can be non‐covalently modified using appropriate counterions. Ion pairing methods and their role in steering drug absorption and transport have been an ongoing topic of research in the pharmaceutical sciences for several decades. Expanding from the findings on small molecules,^[^ ^124^, ^125^ ^]^ researchers made strong efforts to adopt the methods to macromolecules, leading to valuable insights and advancements in biotechnology.^[^ ^126^, ^127^ ^]^ Subsequently, the great potential of hydrophobically modifying peptides, proteins, oligonucleotides, and polysaccharides for the formulation of new drug carrier systems was recognized. Due to the lipid‐based or hydrophobic nature of these carriers, they are natively unable to host hydrophilic biomolecules. Hydrophobic ion pairing presents an efficient way to enable their encapsulation. The rapidly growing market of biopharmaceuticals and great scientific interest ultimately led to the widespread application of hydrophobic ion pairing methods in today's landscape of drug delivery.^[^ ^128^ ^]^

As macromolecular drugs are hydrophilic, water‐soluble, and prone to degradation, their formulation remains challenging. Abundant ionizable functional (e.g., acidic/basic amino acids in peptides and proteins or the polyphosphate backbone of oligonucleotides) natively contribute to the hydrophilicity of these molecules. However, they also serve as the gateway for hydrophobic ion pairing, as they can be modified by oppositely charged ions. Exchange of small, easily solvated counter ions (e.g., Cl^−^ or Na^+^) by bulky and hydrophobic counter ions leads to the formation of HIP with substantial increases in lipophilicity.^[^ ^126^ ^]^ Within HIP, the charged moieties are shielded from being solvated by water molecules, substantially reducing aqueous solubility. Additionally, the bulky residue of the counterions creates a nonpolar shell around the complex.^[^ ^126^, ^127^ ^]^ This lipidation allows for incorporation – most commonly of the isolated precipitate but also in situ^[^ ^129^ ^]^ – into lipophilic carrier systems. For a comprehensive review on the ion pairing process, its optimization, and the incorporation of HIP into different carrier‐systems we refer to several overview articles.^[^ ^128^, ^130^, ^131^ ^]^ Furthermore, interesting studies have been conducted by Wibel et al. comparing different preparation methods for HIP, regarding precipitation efficiency and lipophilicity increase. The Bligh–Dyer method showed the highest lipophilicity increase, additionally allowing for the use of water‐insoluble surfactants.^[^ ^132^ ^]^ Simulation studies by Kozuch et al. investigated the molecular mechanisms of the ion pairing process, optimizing hydrophobicity and cluster morphology of Polymyxin B:oleic acid systems.^[^ ^133^ ^]^

For the hydrophobic counterions, surfactants have emerged as auxiliaries of choice. According to the nature of the functional groups on the drug, the formulation scientist can choose from a broad range of different surfactants to optimize HIP formation and lipophilicity. Regarding the lipophilicity assessment of HIP, the most common approach is represented by the determination of the 1‐octanol/water distribution coefficient (logP). If standard logP procedures are not applicable, also modified protocols are also being used, e.g., distribution between 1‐butanol/water.^[^ ^134^ ^]^ Generally, surfactants with higher logP values are utilized, as the lipophilicity of the HIP will increase accordingly.^[^ ^128^ ^]^ On top of that, by careful selection of other key parameters, like the hydrophilic head properties (e.g., type of functional group and pKa), or the constitution of the hydrophobic chain (e.g., length, branching, and flexibility) hydrophobization can be further improved. Claus et al. investigated the key factors of counterion optimization, establishing the polar head‐group order *sulfonate > sulfosuccinate > phosphate = sulfate > carbonate > phosphonic acids = sulfobetaines*.^[^ ^135^ ^]^ As the hydrophobic ion pairing is driven by electrostatic attraction, highly electron‐dragging and low pKa head‐groups are advantageous, bearing a steady and strong anionic charge.^[^ ^128^, ^131^, ^135^, ^136^ ^]^ An overview and comparison of surfactant head‐groups is depicted in **Table** **5**.

**Table 5:** Representative surfactant head group structures, examples, and features relevant to hydrophobic ion pairing.

Head structure	Example	Features
Sulfate 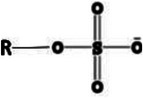	Sodium dodecyl sulfate Sodium tetradecyl sulfate N‐octadecyl sulfate Sodium cholesteryl sulfate Sodium stearyl sulfate	Strong acid (R–OSO_3_^−^), moderate to high toxicity in biological systems, strong electrostatic binding; risk of irritation
Sulfonate 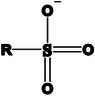	Sodium decane sulfonate Sodium dodecyl benzenesulfonate Sodium docusate Sodium taurodeoxycholate Sodium 1‐butanesulfonate Sodium 1‐heptanesulfonate	Strong acid (R–SO_3_^−^), often safer than sulfates; tauro‐conjugates are endogenous bile salts, better solubility in water; used for both small molecules and biologics
Phosphate 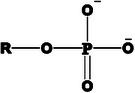	Hexadecyl phosphate Sodium tripoly phosphate Bis(2‐ethylhexyl) phosphate Dibenzyl hydrogen phosphate Dicetyl phosphate Potassium cetyl phosphate	Moderate to strong acid (R–OPO_3_^2−^), generally safe, especially endogenous phosphates, good for mimicking biological molecules; can be multivalent
Carboxylate 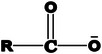	Oleic acid Pamoic acid Sodium stearate Sodium deoxycholate Taurocholic acid Sodium stearoyl glutamate	Weak acid (R–COO^−^), low toxicity, good biocompatibility, especially fatty acids, easily metabolized; useful for biodegradable formulations
Ammonium 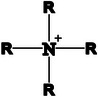	Hexadecyl trimethylammonium Tetrabutyl ammonium bromide Tetraheptyl ammonium bromide Tetrahexyl ammonium bromide Tetracotyl ammonium bromide Tetrapentyl ammonium bromide	Strong base (R_4_N^+^), can be cytotoxic, especially long‐chain alkyl types, strong binding to anionic drugs; surfactant action; cationic surface active agents

**Figure 3:**
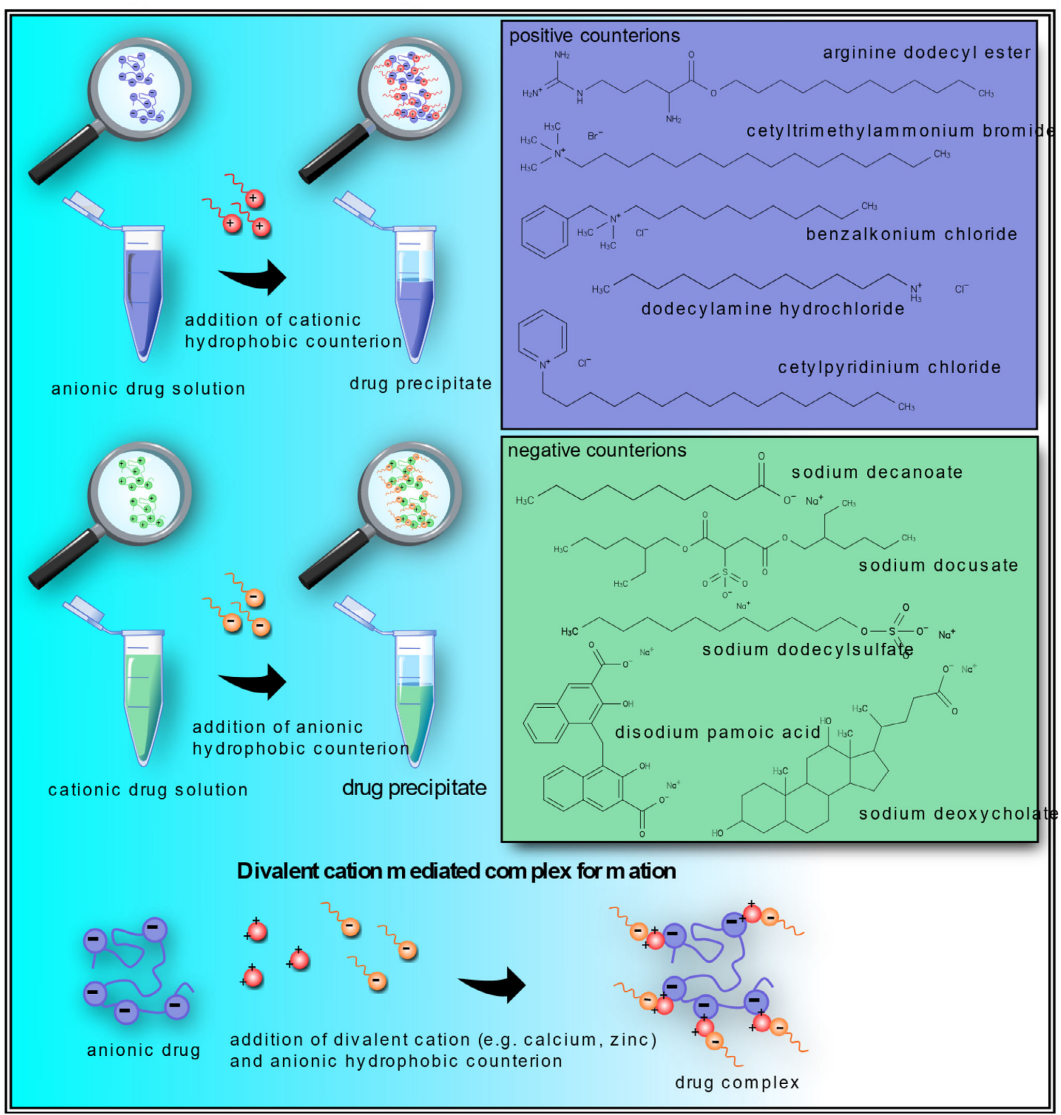
Schematic illustration of hydrophobic ion pairs and their common counterions.

Regarding the hydrophobic moiety, flexible, long, and branched chains showed the highest increase in lipophilicity.^[^ ^135^ ^]^ This can be attributed to enhanced shielding effects covering the hydrophilic surfaces of the protein.^[^ ^137^ ^]^ Most commonly, HIP with peptides/proteins is formed with the drug at a positive state of charge, thus employing anionic surfactants (**Figure** **3**). However, anionic macromolecules can also be paired with anionic surfactants, mediated by divalent cations, as shown for insulin and the LWMH enoxaparin.^[^ ^138^ ^]^ In **Table** **6**, exemplary studies on HIP with peptides/proteins are summarized, comparing the increase in logP and, if available, advancement in bioavailability.

**Table 6:** Exemplary studies improving the lipophilicity of model peptide/protein therapeutics via hydrophobic ion pairing. *Values approximated from the data figure.

Model drug	Surfactants	logD before / after	Formulation	Bioavailability	Biological effect	Reference
Bovine serum albumin (BSA)	Sodium docusate Dodecylsulfate Myristylsulfate	−2.4 / 2.5 (butanol/water)	Self‐emulsifying drug delivery system (SEDDS)	22.2‐fold increase in anti BSA‐IgA serum titer (to oral BSA solution)	Enhanced systemic immunogenicity after oral vaccination with SEDDS, and stronger intestinal IgA response compared to subcutaneous vaccination.	[139]
BSA	Sodium deoxycholate Sodium dodecanoate Sodium stearoyl glutamate Disodium pamoic acid salt (PAM)	−1.0 / 1.0^*^ (PAM) (butanol/water)	SEDDS	–	Incorporation into SEDDS showed controlled release and protection against dissociation.	[134]
Daptomycin	Arginine‐nonyl‐ester (ANE) Arginine‐hexadecyl‐ester (AHE)	−4.5 / 0.5^*^ (AHE)	–	–	Biodegradable and safe alternatives to conventional cationic surfactants with markedly reduced cytotoxicity.	[140]
Daptomycin	Dodecylamine	−5.0 / 4.8	SEDDS	–	Incorporation into SEDDS with high peptide payload, controlled release, enhanced mucus permeability and protection against degradation.	[141]
Desmopressin	Sodium docusate	−6.1 / 0.3 (Capmul 907P/water)	SEDDS	–	In vitro protection from glutathione and α‐chymotrypsin degradation.	[142]
Desmopressin	Sodium docusate	−2.8 / 0.2^*^	SEDDS	–	Incorporation into SEDDS with high peptide payload.	[143]
Insulin	Sodium lauryl sulfate	− 2.6 / 2.6 (pH 2.5)	–	–	HIP complexation preserved insulin's native structure and maintained in vivo blood glucose lowering comparable to free insulin after i.v. administration in rats.	[144]
Insulin	Sodium oleate	−3.0 / 0.0 (pH 4.0)	PLGA nanoparticle (composite microcapsules)	11.5 / (15.6) % oral relative bioavailability compared to s.c. insulin	Microcomposite system for oral insulin delivery, providing sustained blood‐glucose reduction over 24 h after a single dose.	[145, 146, 147]
Insulin	Sodium deoxycholate	0.3 / 1.9	PCL‐PEG polymerosomes	After s.c. injection equivalent to 5 IU insulin kg^−1^ body weight serum insulin values of 64.15 ± 13.28 mIU mL^−1^	Enhanced pharmacokinetics and sustained blood glucose lowering over 8 h in diabetic mouse model.	[148]
Insulin	Sodium deoxycholate	− 2.4 / 0.0	PLGA nanoparticles	11.7% oral relative BA (to S.C.)	Complexes retained in vitro and in vivo activity, enabling oral absorption with sustained hypoglycemia over 24 h in diabetic rat model.	[149]
Insulin	Sodium docusate	−1.0 / 1.9^*^	SEDDS	–	Incorporation into SEDDS with high peptide payload.	[143]
Insulin	Sodium deoxycholate Sodium dodecanoate Sodium stearoyl glutamate Pamoic acid disodium (PAM)	−1.1 / 1.3^*^ (PAM) (butanol/water)	SEDDS	–	Incorporation into SEDDS showed controlled release and protection against dissociation.	[134]
Insulin	Sodium docusate (SD) Sodium dodecyl sulfate (SDS) Sodium dodecylbenzenesulfonate (SDBS)	−1.5 / 3.6 (SD) −1.5 / 3.4 (SDS) −1.5 / 3.5 (SDBS)	SEDDS	Up to 2.52% relative pharmacological activity (to s.c.)	Enhanced permeability (up to 3‐fold) in non‐everted gut sac model and hypoglycemic effect over 8 h in in situ instillation study.	[150]
Insulin glargine	Bis(isotridecyl)sulfosuccinate (BIS) Pamoic acid disodium salt Sodium taurocholate Sodium lauryl sulfate Sodium dodecylbenzenesulfonate	−2 / 2.75 (BIS)	SEDDS	2.13% oral bioavailability for polyglycerol‐SEDDS 1.15% oral bioavailability for PEG‐SEDDS	Sustained oral insulin delivery with controlled glucose‐lowering effect over 6 h in rats.	[151]
Leuprolide acetate	Sodium docusate	−1.8 / 2.8^*^	SEDDS	–	Incorporation into SEDDS with high peptide payload.	[143]
Leuprolide acetate	Sodium deoxycholate Sodium dodecanoate Sodium stearoyl glutamate Disodium pamoic acid salt (PAM)	−1.5 / 1.9^*^ (PAM) (butanol/water)	SEDDS	–	Incorporation into SEDDS showed controlled release and protection against dissociation.	[134]
Melittin	Sodium dodecyl sulfate	−0.3 / 1.5 (pH 7.0)	PLGA nanoparticles	–	Incorporation into nanoparticles with retained growth inhibitory activity in MCF‐7 breast cancer cells.	[152]
Myr‐NT	Sodium dodecyl sulfate (SDS) Sodium dodecylbenzenesulfonate (SDBS) Sodium docusate (DS)	−1.9^*^ / 2^*^ (SDS) −1.9^*^ / 1.5^*^ (SDBS) −1.9^*^ / 1.9^*^ (DS)	SEDDS	–	Enhanced cellular uptake and intracellular distribution of Myr‐NT, resulting in pronounced anti‐proliferative and anti‐invasive effects in MYC‐dependent cancer cells.	[153]
Salmon calcitonin	Bis(isotridecyl) sulfosuccinate	−2.0^*^ / 3.0	SEDDS	Oral relative pharmacological activity of 13.6% (to s.c.)	SEDDS‐incorporated complexes exhibited improved stability in biorelevant media and significant serum calcium level reduction after oral administration in rats.	[154]
Vancomycin	Linoleic acid	−1.4 / 1.4	Solid lipid nanoparticles (SLN)	In vitro antibacterial effect against S. aureus and MRSA strains for 72 h (vancomycin‐HCl: 18 h)	Enhanced antibacterial effect against S. aureus and MRSA strains for 72 h with significant lowering of MIC from 500 to 15.62 µg mL^−1^ compared to free vancomycin.	[155]
Vancomycin	Sodium docusate	1.4‐unit increase	SEDDS	–	3.3‐fold increase in mucus permeability maintaining high cytocompatibility.	[156]

Although less common, positively charged surfactants – mainly quaternary amines like cetyltrimethylammonium bromide (CTAB) – are also efficient to precipitate macromolecules but generally show higher toxicity.^[^ ^131^, ^157^ ^]^ Nevertheless, this is of high importance for the formulation of RNA/DNA drugs with the anionic polyphosphate backbone. Currently, only the ionizable cationic lipids DLin‐MC3‐DMA (MC3), ALC‐0315, and SM‐102 are clinically approved for use in RNA‐based therapeutics.^[^ ^158^ ^]^ These cationic lipids are considered biodegradable; however, only the fatty alcohol or fatty acid substructures are cleaved during degradation, leaving behind the lipophilic tertiary amine. This residual moiety raises ongoing safety concerns. Researchers therefore investigated novel surfactants with lower cytotoxicity, for example, biodegradable, non‐permanently charged arginine or lysine esters.^[^ ^140^, ^157^, ^159^ ^]^ Wolf et al. synthetized the hexadecyl ester of lysine (HL), exhibiting no toxic effect to Caco‐2 cells in concentrations up to 250 µg mL^−1^.^[^ ^159^ ^]^ Due to the rapidly growing interest in gene therapy‐related drugs there is a high need for advanced drug carriers in this field.^[^ ^160^ ^]^ An overview of exemplary studies utilizing HIP as a promising tool to deliver DNA/RNA therapeutics is provided in **Table** **7**. Additional insights into lipophilic DNA/RNA complexes are available in earlier review articles dedicated to this topic.^[^ ^161^, ^162^ ^]^

**Table 7:** Exemplary studies improving the lipophilicity of model DNA/RNA therapeutics via hydrophobic ion pairing.

DNA/RNA	Surfactants	logD before / after	Formulation	Effect	Reference
SARS‐CoV‐2 receptor‐binding domain mRNA (*Pfizer‐BioNTech)*	ALC‐0315	–	Lipid nanoparticles	High vaccine efficacy in clinical trials	[165]
SARS‐CoV‐2 receptor‐binding domain mRNA (*Moderna*)	SM‐102	–	Lipid nanoparticles	High vaccine efficacy in clinical trials	[165]
Cystic fibrosis transmembrane conductance regulator (CFTR) gene pDNA	Cholest‐5‐en‐3‐ol (3β)‐, 3‐[(3‐aminopropyl) [4‐[(3‐aminopropyl)amino]butyl]carbamate] (GL67); 1,2‐dioleoyl‐sn‐glycero‐3‐phosphoethanolamine (DOPE); and 1,2‐dimyristoyl‐sn‐glycero‐3‐phosphoethanolamine‐n‐[methoxy (polyethylene glycol 5000)] (ammonium salt) (DMPE‐PEG5000)	–	Liposomes	Improvement in lung function and bronchial CFTR function, compared with placebo	[166]
Tumor suppressor gene TUSC2/FUS1 (TUSC2) pDNA	N‐[1‐(2,3‐dioleoyloxy)propyl]‐N,N,N‐trimethylammonium chloride (DOTAP)	–	Lipid nanoparticles	Uptake of the gene by human primary and metastatic tumors in lung cancer patients	[167]
pcDNA3‐EGFP	Benzalkoniumchloride Dodecyltrimethylammonium bromide Cetyltrimethylammoniumbromid (CTAB) Cetylpyridiniumchloride	−1.64/2.08 (CTAB)	SEDDS	7.2‐fold increase in EGFP expression after transfection of HEK293 cells	[38]

Despite of their proteinaceous nature, hydrophobic ion pairing with antibody drugs faces some additional challenges in comparison to other protein drugs. Therefore, the limited literature on this topic is addressed separately in this section. Patel et al. highlight the intricate tertiary structure of this high molecular weight drug class, bearing a high density of charged amino groups, impeding the HIP formation.^[^ ^163^ ^]^ They performed a fundamental study on ion pairing of a human IgG‐Fab fragment with sodium dodecylsulfate and taurocholic acid and were able to incorporate the complex into poly‐lactid‐(co‐glycolid) (PLGA) nanoparticles.^[^ ^163^ ^]^ In 2024, Pangua et al. published a study on HIP with the monoclonal antibody Bevacicumab, preparing HIP with sodium deoxycholate and sodium docusate. Incorporated into PEG‐coated albumin nanoparticles, they showed a 1000‐fold increase in bioavailability in rats to 3.7%, compared to non‐ion‐paired drug.^[^ ^164^ ^]^ Analogous to the antibodies, there are only few studies addressing HIP with polysaccharide drugs. However, some studies successfully prove the potential of HIP also in this field. An overview is provided in **Table** **8**, with studies focusing mainly on HIP with heparin and its derivatives.

**Table 8:** Exemplary studies improving lipophilicity of model polysaccharide therapeutics via hydrophobic ion pairing. *Values approximated from data figure.

Drug	Surfactant/System	logD before / after	Formulation	Bioavailability	Reference
Enoxaparin (MW 4500 Da)	Benzalkoniumchloride Cetyltrimethylammoniumbromide Dodecylamine hydrochloride (DOA)	–	SEDDS	2.25% (DOA)	[168]
Heparin (MW 16500 Da)	Arginine‐nonyl‐ester Arginine‐hexadecyl‐ester	−7.5 / 0.3^*^	–	–	[140]
Enoxaparin (MW 4500 Da)	Cetyltrimethylammoniumbromide	–	PLGA nanoparticles	–	[169]
Enoxaparin (MW 4500 Da)	Dodecyltrimethylammonium bromide Didecyldimethylammoniumbromide Dodecylamine hydrochloride Dioleoyl‐3‐trimethylammonium propane Tetraheptylammoniumbromide	–	Nanoemulsions	2.6%	[170]

Abstracting from all advancements in the respective fields of macromolecular drug delivery via hydrophobic ion pairing, this method stands out as a promising pathway for innovative drug carrier design. Recent insights into counterion optimization and the synthesis of avant‐garde surfactants have greatly improved performance, particularly in terms of payload and oral bioavailability. Furthermore, the assistance of molecular modelling offers a deeper understanding of the complex optimization process, paving the way for more precise, efficient and targeted drug delivery in the future.

### 3.2 Reverse Micelles

**Figure 4:**
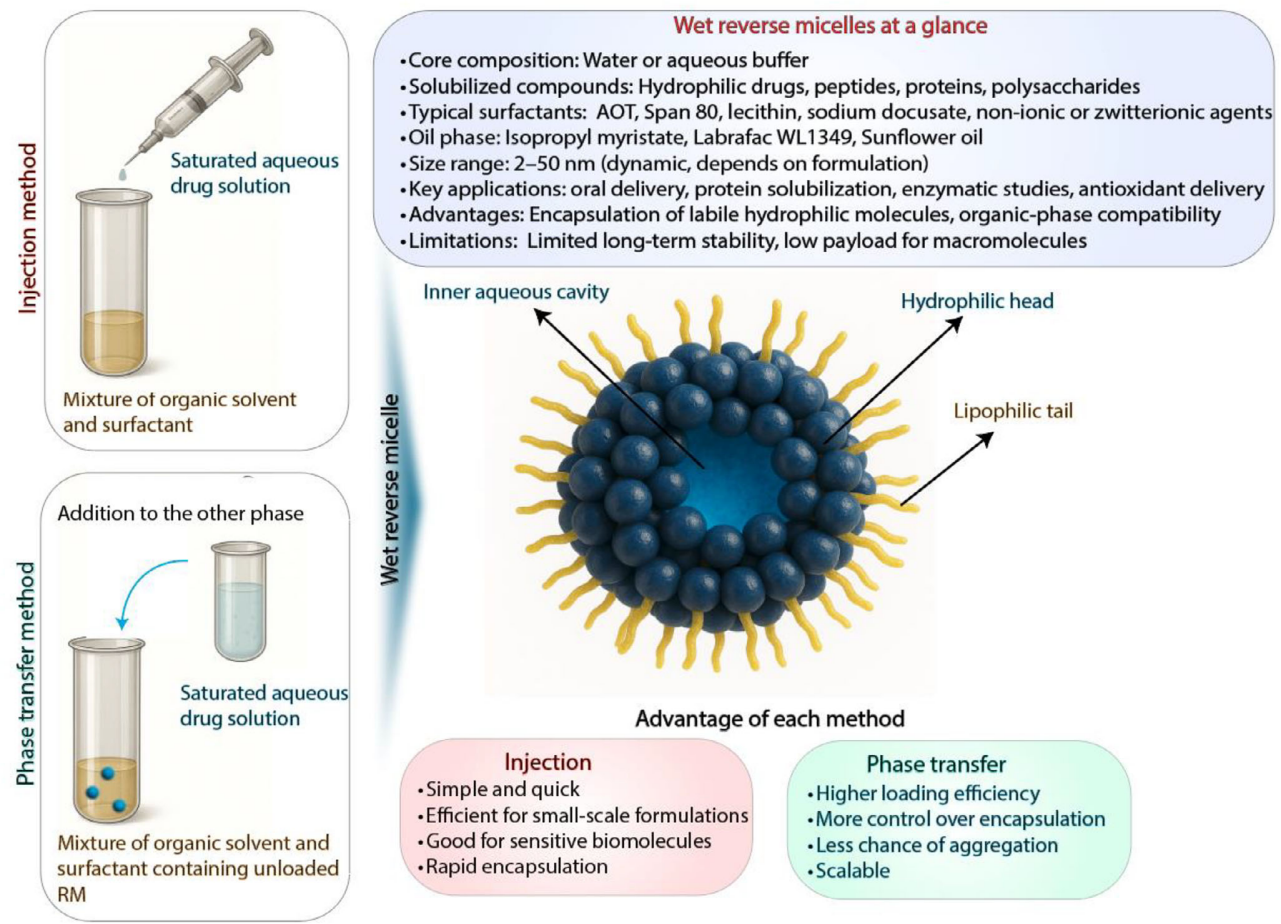
Overview of wet RM and their characteristics, advantages, and limitations.

**Figure 5:**
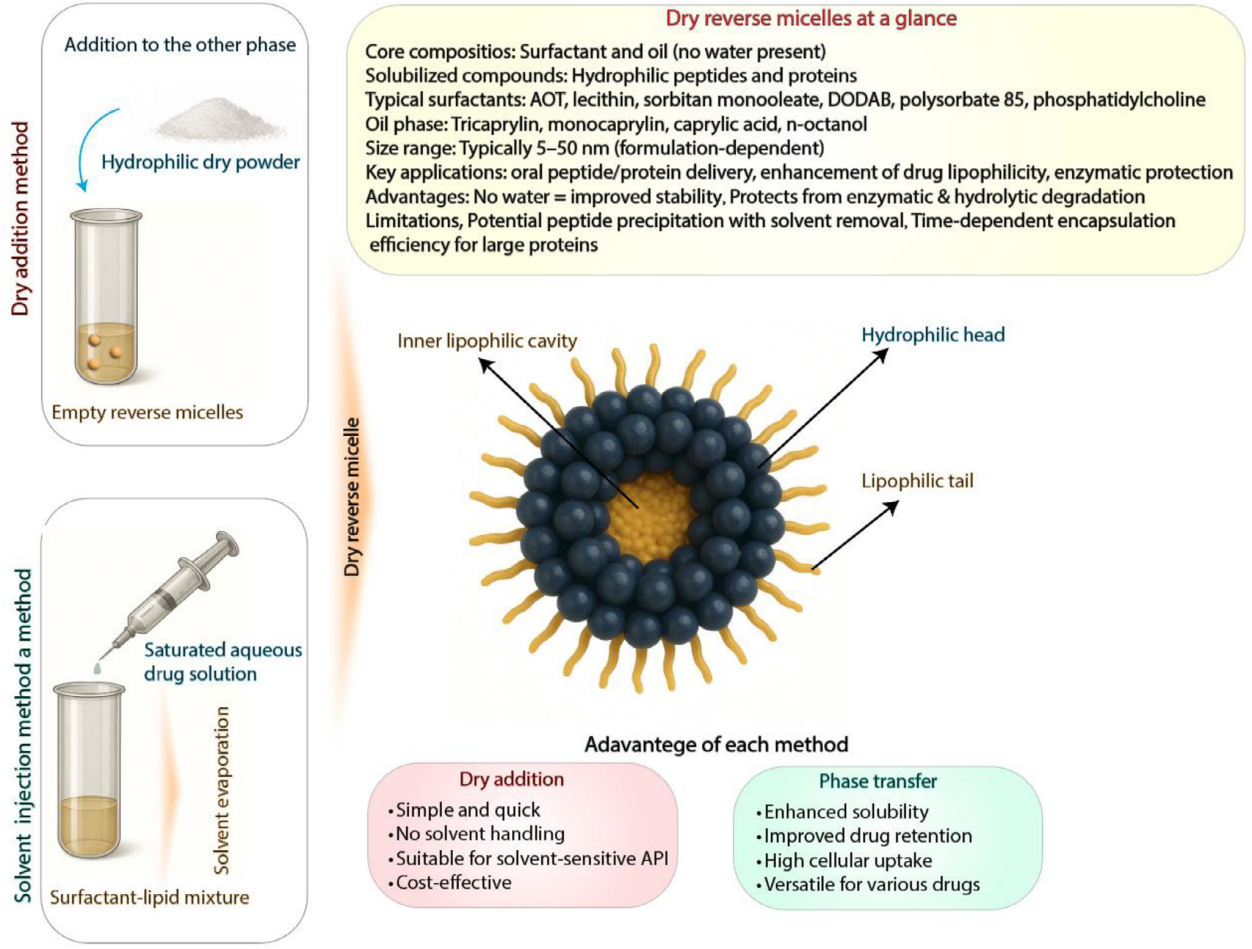
Overview of dry RM and their characteristics, advantages, and limitations.

Reverse micelles (RM) are nanoscale structures formed by a monomolecular surfactant layer dispersed in a non‐polar solvent. In these systems, the lipophilic chains of the surfactants orient outward into the non‐polar bulk, while the polar head groups face inward, creating a polar core. This structure allows for the incorporation of hydrophilic substances in non‐polar environments, useful for applications such as protein extraction,^[^ ^171^, ^172^, ^173^ ^]^ nano‐reactors for enzymatic reactions^[^ ^174^ ^]^ and enhanced drug delivery for hydrophilic drugs.^[^ ^175^ ^]^ They can be prepared using various types of surfactants: anionic, cationic, and non‐ionic. Ionic surfactants primarily interact through electrostatic forces, with additional contributions from hydrophobic and steric hindrance interactions.^[^ ^176^ ^]^ Non‐ionic surfactants, on the other hand, interact through hydrophobic and hydrogen bonding.^[^ ^177^ ^]^ Furthermore, mixed RM can be created by combining anionic or cationic surfactants with non‐ionic surfactants.^[^ ^178^ ^]^ RM are categorized into wet (**Figure** **4**) and dry (**Figure** **5**) types,^[^ ^179^ ^]^ each offering specific advantages for enhancing the lipophilicity of macromolecular drugs.

#### 3.2.1 Wet Reverse Micelles

**Figure 6:**
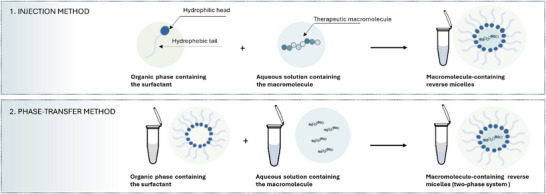
Schematic illustration of wet reverse micelle preparation methods.

Wet RM are characterized by their aqueous inner core, essential for the encapsulation and solubilization of hydrophilic substances.^[^ ^180^ ^]^ The lipophilicity of hydrophilic macromolecular drugs is consequently not directly increased, as they are still surrounded by an aqueous environment. Nevertheless, this approach enables the incorporation of hydrophilic drugs within non‐polar environments. The size of these RM is controlled by the water‐to‐surfactant ratio, with larger ratios resulting in an increased radius.^[^ ^178^ ^]^ Two primary methods are used to prepare wet RM: the injection method and the phase‐transfer method (**Figure** **6**). In the injection method, a saturated aqueous drug solution is added to a mixture of surfactant and organic solvent, followed by vigorous mixing, resulting in the formation of RM around the drug solution.^[^ ^181^ ^]^ The phase‐transfer method involves a forward and backward extraction process; the organic phase containing RM and the aqueous phase containing the hydrophilic drug are mixed and centrifuged to obtain a two‐phase system, where the upper organic phase contains loaded RM.^[^ ^182^ ^]^ In addition to choosing the appropriate surfactant and oil, other parameters such as ionic strength, phase ratio, pH of the aqueous phase, and temperature are crucial for developing wet reverse micelles, particularly when using the phase‐transfer method.

The literature provides several examples of wet reverse micelles (RM) encapsulating hydrophilic peptides, prepared using the injection method. For instance, the antidiabetic drug exenatide was solubilized into RM containing Span 80 in Labrafac WL1349 by injecting the drug solution into the surfactant‐oil mixture, followed by high‐speed stirring.^[^ ^183^ ^]^ Loaded RM were subsequently encapsulated in lipid nanocarriers to enhance mucus permeability and improve intestinal absorption. Additionally, utilizing Labrafac WL1349 as the oil phase, RM comprising sodium docusate was developed to encapsulate the antimicrobial peptide AP138, effective against gram‐positive bacteria.^[^ ^175^ ^]^ However, these studies primarily focused on the formulation properties, thus the RM encapsulating the peptides was not further characterized and assessed. Stackhouse et al. studied unconventional surfactants for wet RM formation.^[^ ^184^ ^]^ They found that a mixed system of 1‐decanoyl‐*rac*‐glycerol and lauryldimethylamine‐N‐oxide solubilized model proteins with more stability than traditional spherical RM systems like dioctyl sulfosuccinate sodium (AOT) or CTAB, due to their elliptical shape. This form allowed a core with more bulk‐like water, effectively solubilizing hydrophilic proteins such as cytochrome c, myoglobin, and flavodoxin via direct injection.

Compared to RM solubilizing peptides and proteins, there is limited research available on RM encapsulating other hydrophilic macromolecules besides peptides. Ehsandoost et al. examined the antioxidant effect of the polysaccharide fucoidan in sunflower oil by incorporating it into lecithin wet RM.^[^ ^185^ ^]^ The maximum tested concentration of fucoidan, a large hydrophilic polysaccharide of 140 kDa with a measured LogP of −1.49, was 0.08% (w/w). As a result, lecithin‐RM not only increased the solubility of fucoidan in sunflower oil but also synergistically enhanced its antioxidant effect.

#### 3.2.2 Dry Reverse Micelles

**Figure 7:**
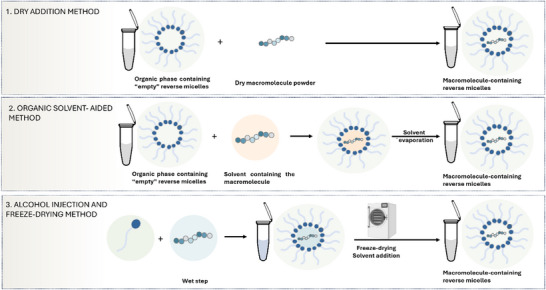
Schematic illustration of dry reverse micelle preparation methods.

Dry RM is composed of surfactants and oils but does not contain water in its core.^[^ ^186^ ^]^ They are often preferred over wet RMs as they protect peptide drugs from enzymatic cleavage,^[^ ^187^ ^]^ hydrolytic degradation during storage, or interaction with endogenous compounds upon administration.^[^ ^188^ ^]^ The formation of dry RM has been studied using different methods (**Figure** **7**). Recently, the dry addition method and organic‐solvent aided injection method were compared for their drug solubilization ability and increase in lipophilicity. In the dry addition method, hydrophilic model drugs, like the antibiotic peptide bacitracin, were directly added to empty reverse micelles. In the solvent‐aided method, drugs were dissolved in water or organic solvents, then injected into a surfactant‐lipid mixture, followed by solvent evaporation.^[^ ^188^ ^]^ For RM comprising AOT and the zwitterionic phosphatidylcholine, high bacitracin solubilities were achieved using the organic solvent‐aided method. Specifically, a 97‐fold increase in solubility was observed with AOT‐comprising RM compared to the same RM prepared by the dry addition method. An even higher solubility, up to 35.2 mg mL^−1^, was obtained with RM containing sorbitan monooleate. Notably, RM with sorbitan monooleate exhibited greater bacitracin solubilizing abilities when prepared with the dry addition method. It is hypothesized that bacitracin's limited solubility in organic solvents, due to its high hydrophilicity, leads to peptide precipitation upon water evaporation.

Using the dry addition method, Jörgensen et al. developed dry RM incorporating the model peptide polymyxin B.^[^ ^179^ ^]^ This study was among the first to investigate dry RM with a peptide drug for oral peptide delivery, demonstrating the potential of these systems for hydrophilic macromolecular drugs. Representative anionic (AOT), cationic (dimethyl dioctadecyl ammonium bromide (DODAB)), zwitterionic (lecithin), and non‐ionic (polysorbate 85) surfactants for RM formation were evaluated and compared in terms of their ability to solubilize polymyxin B and enhance its lipophilicity. Lecithin‐RM solubilized the highest amount of polymyxin B (up to 20.01 mg mL^−1^), while AOT yielded impressively high LogD_lipophilic phase/water_ values of almost 3. DODAB demonstrated the most potential concerning cellular uptake and drug absorption when further formulated into lipid‐based nanocarriers. Cellular uptake studies using Caco‐2 cells revealed that formulations with DODAB‐RM exhibited ≈100% cellular uptake, likely due to the cationic nature of DODAB, facilitating strong interactions with negatively charged cell membranes. Sandmeier et al. successfully incorporated various therapeutic and model peptides and proteins into dry RM using the dry addition method.^[^ ^189^ ^]^ They solubilized colistin (1.1 kDa), semaglutide (4.1 kDa), lysozyme (14.4 kDa), and bovine serum albumin (BSA) (66.4 kDa) in RM using AOT and ethyl lauroyl arginate. They discovered that hydrophilic macromolecule solubilization in RM is time‐dependent, with higher solubility after longer incubation (e.g., 48 h). A correlation between molecular size and encapsulation was noted, with semaglutide showing 10‐fold higher solubility than lysozyme, while BSA achieved solubilities of less than 1 mg mL^−1^ even after prolonged incubation.

On the other hand, the combination of alcohol injection and freeze‐drying technology, which results in the production of dry RM (commonly referred to as anhydrous reverse micelles (ARM) in literature), demonstrates significant potential in enhancing the lipophilicity of hydrophilic peptides and proteins. This method involves the initial mixing of an aqueous phase containing hydrophilic macromolecules with an oil phase containing lecithin, followed by lyophilization. The resultant complex is then dissolved in an oil phase or encapsulated into polymer‐based microspheres for administration. Various peptide drugs, such as insulin,^[^ ^190^, ^191^ ^]^ salmon calcitonin,^[^ ^192^ ^]^ along with model proteins like BSA^[^ ^193^ ^]^ and vascular endothelial growth factor (VEGF),^[^ ^194^ ^]^ have been successfully incorporated into dry RM composed of lecithin. Specifically, the solubility of insulin in a lipid mixture of tricaprylin and monocaprylin was significantly enhanced to 1.4 mg mL^−1^ using phosphatidylcholine RM.^[^ ^190^ ^]^ These RM effectively protected insulin from enzymatic degradation, achieving bioavailability of up to 5.6% in diabetic rats, which is notably higher than that of orally administered native insulin. Additionally, dry RM for salmon calcitonin reduced administration frequency due to longer circulation and improved therapeutic effects compared to the native peptide, reducing osteoporosis symptoms in glucocorticoid‐induced osteoporosis mice.^[^ ^192^ ^]^ Using a similar preparation method, Ricci et al. incorporated a larger macromolecule, the model protein horseradish peroxidase (HRP), into dry RM.^[^ ^195^ ^]^ Unlike other studies, this research utilized not only phosphatidylcholine as a surfactant but also mixed dry RM with AOT. The combined effect of the two surfactants resulted in HRP solubility of 3% (w/w) in the dry RM, which was more than 15 times higher than in other studies.^[^ ^196^ ^]^ Additionally, lipophilicity increased significantly, with a LogP_n‐octanol/water_ of 3.1 for the dry RM compared to −3.36 for native HRP. Furthermore, the LogD_caprylic acid/water_ reached 4.01, whereas the native protein was undetectable in the oil. In comparison, HIPs with HRP and AOT as the counterion only increased the lipophilicity of HRP to a LogD_n‐butanol/water_ of 1.3. Measurement in less polar n‐octanol was not feasible as the resulting complex was not soluble in n‐octanol. Additionally, incorporating HRP into dry RM protected it from enzymatic degradation, with more than 95% of the protein remaining intact.

Overall, RM represents a highly versatile platform for enhancing the solubility, lipophilicity, and stability of hydrophilic macromolecular drugs. Both wet and dry RM systems offer distinct advantages depending on the therapeutic application, with dry RM particularly excelling in protecting sensitive biomolecules from enzymatic degradation. Continued optimization of RM formulations holds a significant promise for advancing the oral delivery and bioavailability of hydrophilic peptides, proteins, and polysaccharides.

## 4 Comparison of Lipidation Strategies

Both covalent and non‐covalent lipidation strategies play important roles in enhancing the lipophilicity of macromolecular therapeutics, thereby improving their absorption, stability, and bioavailability. Covalent lipidation involves the chemical attachment of lipids (such as fatty acids or steroids) to the therapeutic molecule, whereas non‐covalent approaches, like hydrophobic ion pairing and RM formation, rely on reversible interactions with lipophilic surfactants. Due to these fundamentally different mechanisms – permanent covalent bonds versus reversible non‐covalent interactions – each approach is suited to distinct applications.

Covalent lipidation offers the advantage of a stable and durable lipid attachment that is not influenced by environmental conditions upon administration. Moreover, it allows for additional chemical tailoring through the selection of specific linkers and modifications. For instance, in the case of insulin icodec, the introduction of a spacer enhanced albumin binding, contributing to its exceptionally long half‐life of 196 h.^[^ ^75^ ^]^ As detailed in this review, covalent lipidation not only improves pharmacokinetics but also facilitates membrane permeability, leading to the successful commercialization of lipidized macromolecular drugs. However, because covalent modification creates new active pharmaceutical ingredients (API) with altered structures and potentially altered biological activities, obtaining regulatory approval can be more complex and demanding.

An additional critical consideration is that covalent lipidation may also alter the immunogenicity profile of macromolecular drugs. In the context of vaccine development, this effect is often intentional. For instance, Zeng et al. demonstrated that conjugation of the TLR2 agonist Pam_2_Cys to insulin markedly enhanced its immunogenicity.^[^ ^197^ ^]^ By contrast, unintended immune activation is a major concern in therapeutic development. Immunogenicity can be triggered when structural modifications generate neoepitopes that antigen‐presenting cells present via MHC class II, thereby priming T‐cell responses.^[^ ^198^ ^]^ Additionally, lipidation‐induced effects such as altered aggregation or albumin binding can influence antigen disposition and immune recognition.^[^ ^199^ ^]^ Importantly, these effects are context‐dependent: structural masking or increased stability can reduce immunogenicity, as illustrated by lipidated GLP‐1 analogues like semaglutide, which exhibit low anti‐drug antibody incidence in clinical trials.^[^ ^200^ ^]^ Therefore, systematic strategies are needed to mitigate immunogenic risks. In silico prediction tools provide a first‐line approach for identifying T‐cell epitopes and evaluating how lipidation may influence immune recognition.^[^ ^201^ ^]^ Engineering strategies such as epitope shielding, sequence optimization or glycosylation tuning are increasingly applied to reduce recognition.^[^ ^202^ ^]^ Another widely used approach is the conjugation of endogenous‐like fatty acids such as palmitate or stearate, which provide half‐life extension through albumin binding while minimizing immunogenic potential. Examples using this strategy are liraglutide (C16) and semaglutide (C18 diacid), where high sequence homology to native GLP‐1 further contributes to their favorable immunogenicity profiles.^[^ ^15^ ^]^ Linker chemistry is another determinant: stable amide linkers can reduce T‐cell activation, whereas thioesters may enhance recognition, depending on their persistence during antigen processing.^[^ ^15^ ^]^ Aggregation control is also critical, as aggregates and self‐assembled lipidated species can act as potent immunogens. Orthogonal analytical methods such as light scattering, size‐exclusion chromatography, and particle counting are recommended to monitor aggregation, and formulation strategies such as pH, ionic strength, and excipient optimization can help to maintain monomeric stability.^[^ ^203^ ^]^ Finally, comprehensive immunogenicity risk assessment is essential throughout development. This involves integrating product‐related risk matrices, predictive in silico/in vitro screening, validated binding and neutralizing anti‐drug antibody assays, and clinical monitoring strategies to correlate anti‐drug antibody presence with pharmacokinetic, pharmacodynamic, safety, and efficacy endpoints.^[^ ^204^, ^205^ ^]^ Together, these approaches provide a framework for balancing the therapeutic benefits of lipidation with its potential immunogenic risks.

In contrast, non‐covalent lipidation strategies such as HIP and RM offer advantages from an industrial and regulatory standpoint. Since these methods do not permanently alter the macromolecule, these drugs fall under abbreviated new drug applications (ANDAs) or 505(b)(2) regulatory approval pathway of the FDA, with fewer clinical and toxicity studies as needed for prodrugs in order to obtain approval.^[^ ^206^ ^]^ Additionally, the native structure and function of the drug are preserved. Preclinical studies have demonstrated that non‐covalent lipidation can similarly improve stability, permeability, and oral bioavailability. Moreover, the dynamic and reversible nature of HIP and RM allows for flexible formulation adjustments, enabling targeted tissue delivery or controlled drug release through the selection of appropriate surfactant partners. Additionally, screening and optimization are facilitated by the relatively simple preparation processes, avoiding the need for complex chemical synthesis. A detailed comparison of the advantages and limitations of both covalent and non‐covalent lipidation strategies is summarized in **Table** **9**.

**Table 9:** Advantages and limitations of covalent and non‐covalent lipidation.

	Covalent lipidation	Non‐covalent lipidation
Reversibility	Irreversible attachment of lipids (e.g., fatty acid, steroids) through chemical modification	Reversible binding of lipids (e.g., surfactants) through electrostatic and/or hydrophobic interactions
Stability	Stable, long‐lasting modification that resists dissociation in circulation due to stable chemical bonds (e.g., amide or ester bonds) between lipids and macromolecules; covalent lipidation can trigger the formation of oligomers and fibrils	Possibility of dissociation and disruption by dilution, interactions with endogenous compounds or changes in the physiological environment due to weaker non‐covalent interactions
Therapeutic activity	Permanent modification might affect the native structure, activity or binding characteristics of the macromolecule, leading to potential alteration of its function	Intrinsic structure and bioactivity are preserved as there is no chemical modification of the macromolecule itself, preserving its function
Drug release	Controlled release possible with, e.g., cleavable linkers (e.g., pH‐sensitive, enzyme‐cleavable) between the attached lipid and the macromolecule	Potential for premature release of the drug before reaching the intended target due to the relatively labile nature of non‐covalent complexes
Formulation	Synthetic complexity as site‐specific conjugation demonstrates challenges, resulting in heterogenous mixtures requiring extensive purification procedures	Simple formulation under mild conditions without the need for complex synthesis and purification processes
Toxicity	New lipid moieties attached to the macromolecules can potentially trigger unwanted immune responses and thus demonstrate immunogenicity risks	The surfactants used might interact with other biomolecules which can lead to non‐specific binding or toxicity issues
Regulatory approval	More complicated as the conjugated macromolecule is regarded as a new API	Potential streamlining of the regulatory approval process as the resulting structure is not regarded as a new API

When comparing HIP and RM specifically, RM systems offer broader applicability, as they are less reliant on the presence of charged groups within the macromolecule, making them suitable for a wider variety of hydrophilic drugs.^[^ ^188^ ^]^ In a comparative study, Sandmeier et al. investigated semaglutide formulations using both dry RM and HIP with the same surfactants.^[^ ^189^ ^]^ The maximal solubility of semaglutide was achieved within 24 h with HIP, compared to over 48 h for RM, though overall solubility was found to be twice as high in RM as in HIP. Importantly, RM also provided greater enhancement in lipophilicity: HIP increased semaglutide's lipophilicity by ≈2.4 × 10⁴‐fold relative to the native peptide (resulting in a LogP below 1), while RM further improved lipophilicity to achieve a LogP above 2. This superior performance of RM can be attributed to the higher number of surfactant molecules associated with each peptide, compared to the charge‐dependent nature of HIP. Furthermore, for larger proteins like lysozyme, RM enhanced lipophilicity by 600‐fold relative to the native molecule and by 7.5‐fold compared to HIP, demonstrating RM's significant potential for improving the delivery of hydrophilic macromolecules.

## 5 Outlook and Conclusion

Lipidation has emerged as a particularly valuable strategy in drug delivery, with several lipidized drugs already available on the pharmaceutical market. By covalently attaching lipophilic moieties, lipidation converts hydrophilic peptides into more lipophilic compounds, thereby enhancing their pharmacological properties. Among the various techniques, fatty acid acylation remains the most widely utilized. The broad range of adaptable chemical reactions applicable to different carrier systems underscores the growing importance of this field – a trend likely to be reflected in the increasing number of lipidized conjugates gaining market access and regulatory approval. We believe the full potential of covalent conjugation in drug delivery systems is only beginning to be realized, understood, and exploited.

While covalent lipidation offers stable and long‐lasting modifications, non‐covalent strategies – such as hydrophobic ion pairing (HIP) and reverse micelle (RM) formation – have shown distinct advantages, particularly in preserving the native structure of macromolecules and streamlining regulatory approval. Among these, RM systems tend to outperform HIPs in enhancing both solubility and lipophilicity, especially for large and structurally complex biomolecules. Their dynamic and tunable nature makes them highly attractive for future pharmaceutical applications.

Although HIPs were first explored in the 1970s and 1980s and demonstrated high membrane permeability in in vitro studies, few in vivo studies have been conducted, and none have advanced to clinical trials. This limited progress is largely due to the poor stability of HIPs under physiological conditions. A study investigating the stability of various HIPs in gastrointestinal fluids found that these complexes dissociate within hours.^[^ ^134^ ^]^ Both HIPs and RM are effective for enhancing the drug delivery of macromolecules, but they remain susceptible to dilution, competition with endogenous molecules, and changes in the physiological environment. One destabilizing factor is displacement by endogenous compounds, particularly serum proteins and bile salts, which can exchange hydrophobic counterions or surfactants. The adsorption of a protein corona can further alter colloidal stability and accelerate clearance, making surface engineering strategies essential to minimize non‐specific interactions.^[^ ^207^ ^]^

To overcome this, delivery systems must be developed that maintain HIP and RM stability in vivo. Lipid‐based nanoparticles, in particular, have shown promise in this regard. Due to the lower dielectric constant of lipid‐based carriers compared to water, HIP stability is significantly increased in such systems. According to

(1) $$F=K\frac{q_{1}\;q_{2}}{r^{2}\varepsilon }$$

Coulomb's law the electrostatic force (F) binding HIPs is influenced by the Coulomb constant (K), the point charges q_1_ and q_2_, the distance between charges (r), and the dielectric constant (ε); consequently, the association constant in lipid phases is ≈10–50 times higher than in aqueous media.^[^ ^130^ ^]^ Furthermore, hydrophilic counterions such as salts are generally unable to penetrate the lipophilic interior of these carriers, preserving the integrity of the complex. Embedding HIPs or RM in lipid nanocarriers, such as solid lipid nanoparticles or self‐emulsifying drug delivery systems (SEDDS), can provide steric and kinetic protection against displacement.^[^ ^208^ ^]^ This design also buffers against changes in ionic strength and provides protection from enzymatic degradation while preserving the uptake and bioactivity of the encapsulated peptide.^[^ ^136^, ^179^ ^]^ Preliminary studies using lipid‐based nanoparticles with HIPs have already demonstrated high efficacy.^[^ ^151^, ^209^ ^]^ For instance, a representative study reported 28% oral bioavailability of exenatide complexed with tetraheptylammonium bromide and incorporated into self‐emulsifying drug delivery systems (SEDDS).^[^ ^210^ ^]^ Thus, embedding HIPs and RM systems into advanced lipid nanocarriers emerges as a promising approach to address the key stability limitations that have so far restricted their clinical translation.

Furthermore, it is worth mentioning that the pulmonary route is another option for non‐invasive delivery of macromolecular therapeutics. Providing a large absorptive surface, thin epithelial barriers, and the ability to circumvent first‐pass metabolism, the lung allows rapid systemic exposure. Recent work underlines that lipid moieties, either through chemical modification or formulation in lipid nanoparticles may enhance stability and residence time in the lung.^[^ ^211^ ^]^ For instance, cetyl palmitate–based lipid nanoparticles carrying the antimicrobial peptide LL‐37 demonstrated favorable pulmonary retention and stability.^[^ ^212^ ^]^ However, while the pulmonary delivery of polymyxins for treatment of respiratory tract infections is investigated in several published articles,^[^ ^213^ ^]^ research on other lipidated peptides administered via the lung remains an emerging field. Accordingly, most research and clinical translation efforts continue to prioritize oral drug delivery of lipidated molecules.

Many surfactants used in HIP and RM systems – such as medium‐chain fatty acids or bile salts – are themselves potent permeation enhancers.^[^ ^214^ ^]^ Thus, these lipophilic complexes inherently possess high membrane permeability. However, the potential synergies between permeation‐enhancing surfactants and lipophilic complexation strategies remain underexplored.

A major limitation of current systems is the toxicity associated with many commonly used surfactants, particularly non‐biodegradable cationic lipids like quaternary ammonium compounds. These permanently charged molecules disrupt cellular and nuclear membranes, induce lysosomal enzyme release, cause mitochondrial dysfunction, generate reactive oxygen species, and even inflict DNA damage.^[^ ^215^ ^]^

Fortunately, biodegradable and less toxic alternatives to conventional surfactants are currently under development.^[^ ^216^ ^]^ Quaternary ammonium surfactants, which are associated with significant toxicity, are expected to be replaced by biodegradable cationic lipids – particularly those derived from naturally occurring amino acids such as arginine, lysine, or histidine. A notable example is ethyl lauroyl arginate, a compound that degrades into non‐toxic, endogenous metabolites – ethanol, lauric acid, and arginine. Already widely used in the food industry, it holds *Generally Recognized as Safe* (GRAS) status. Preliminary studies suggest that ethyl lauroyl arginate and similar arginine‐based cationic lipids can form highly lipophilic complexes with hydrophilic macromolecules, which are well‐suited for incorporation into lipid‐based nanoparticles.^[^ ^189^ ^]^ The adoption of such biodegradable, low‐toxicity excipients represents a major step forward in designing safer and more effective lipophilic drug complexes.

Overall, both covalent and non‐covalent lipidation strategies offer powerful means of enhancing the delivery of macromolecular drugs, each presenting unique advantages and limitations. The selection of an appropriate approach should be guided by specific therapeutic objectives, molecular properties, and regulatory considerations. Continued innovation – particularly in refining non‐covalent systems such as RM – will be critical to fully realizing the potential of lipidation technologies in improving bioavailability and achieving clinical success for macromolecular therapeutics.

However, most current research in this field relies heavily on in vitro and preclinical animal models, leaving the safety, efficacy, and limitations of these strategies in humans largely uncharacterized. The scarcity of robust clinical data remains a significant barrier to the advancement and translation of novel lipidation‐based delivery systems.

Given the inherent complexity of lipidating hydrophilic macromolecules, it is unlikely that a single approach will address all associated challenges. Moving forward, progress will depend on the development of new concepts, innovative design strategies, and advanced experimental models. The integration of emerging technologies – such as artificial intelligence – will play an increasingly important role in accelerating the discovery and optimization of more effective lipidation techniques.

## Conflict of Interest

The authors declare no conflict of interest.

## Author Contributions

The conceptualization of the work was carried out by S.L. and A.B.‐S. The original draft was written by S.L., S.K., S.H., O.F.R., K.Z., G.S., I.C., and K.K. Writing, review, and editing were performed by S.L. and A.B.‐S. Supervision was provided by A.B.‐S.
